# (*E*)-1-Nitro-2-(2-nitro­prop-1-en­yl)benzene

**DOI:** 10.1107/S1600536812029947

**Published:** 2012-07-07

**Authors:** Li-Li Shen, Zhao-Bo Li, Jia-Jia Li

**Affiliations:** a Zhejiang University of Technology, Hangzhou 310014, People’s Republic of China; b State Key Laboratory Breeding Base of Green Chemistry-Synthesis Technology, Zhejiang University of Technology, Hangzhou 310014, People’s Republic of China; c Hangzhou Minsheng Pharmaceutical Group Co. Ltd, Hangzhou 310000, People’s Republic of China; d Hangzhou Radio and Television University, Hangzhou 310000, People’s Republic of China

## Abstract

The title compound, C_9_H_8_N_2_O_4_, adopts an *E* conformation about the C=C bond. The CH_phen­yl_—C_phen­yl_—CH—C(—NO_2_) torsion angle is −57.7 (3)°. The crystal structure features weak inter­molecular C—H⋯O inter­actions.

## Related literature

For background to nitro­alkenes, see: Ballini & Petrini (2004 ▶); Berner *et al.* (2002 ▶); Ono (2001 ▶).
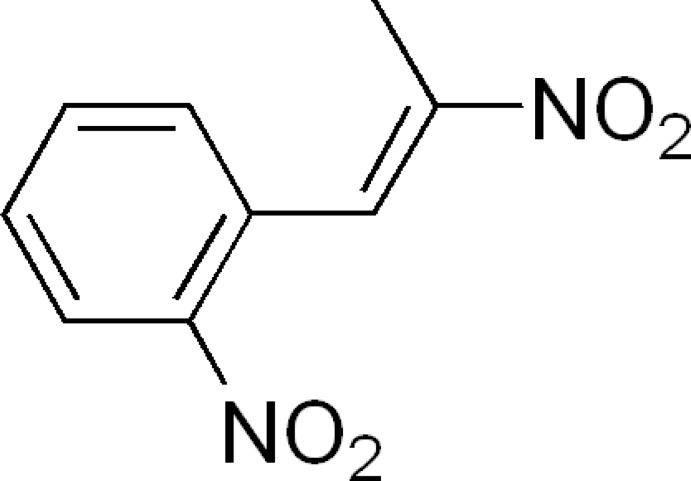


## Experimental

### 

#### Crystal data

C_9_H_8_N_2_O_4_
*M* *_r_* = 208.17
Monoclinic, 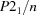

*a* = 6.8274 (9) Å
*b* = 15.5666 (12) Å
*c* = 9.9045 (10) Å
β = 113.202 (3)°
*V* = 967.51 (18) Å^3^
*Z* = 4
Mo *K*α radiation
μ = 0.12 mm^−1^
*T* = 296 K
0.58 × 0.46 × 0.32 mm

#### Data collection

Rigaku R-AXIS RAPID/ZJUG diffractometer
Absorption correction: multi-scan (*ABSCOR*; Higashi, 1995 ▶) *T*_min_ = 0.932, *T*_max_ = 0.964
7450 measured reflections
1736 independent reflections
1193 reflections with *I* > 2σ(*I*)
*R*_int_ = 0.072

#### Refinement

*R*[*F* ^2^ > 2σ(*F* ^2^)] = 0.059
*wR*(*F* ^2^) = 0.135
*S* = 1.00
1736 reflections
138 parameters
H-atom parameters constrained
Δρ_max_ = 0.26 e Å^−3^
Δρ_min_ = −0.21 e Å^−3^

### 

Data collection: *PROCESS-AUTO* (Rigaku, 2006 ▶); cell refinement: *PROCESS-AUTO*; data reduction: *CrystalStructure* (Rigaku, 2007 ▶); program(s) used to solve structure: *SHELXS97* (Sheldrick, 2008 ▶); program(s) used to refine structure: *SHELXL97* (Sheldrick, 2008 ▶); molecular graphics: *ORTEP-3 for Windows* (Farrugia, 1997 ▶); software used to prepare material for publication: *WinGX* (Farrugia, 1999 ▶).

## Supplementary Material

Crystal structure: contains datablock(s) I, global. DOI: 10.1107/S1600536812029947/ng5280sup1.cif

Structure factors: contains datablock(s) I. DOI: 10.1107/S1600536812029947/ng5280Isup2.hkl

Supplementary material file. DOI: 10.1107/S1600536812029947/ng5280Isup3.cml

Additional supplementary materials:  crystallographic information; 3D view; checkCIF report

## Figures and Tables

**Table 1:** Hydrogen-bond geometry (Å, °)

*D*—H⋯*A*	*D*—H	H⋯*A*	*D*⋯*A*	*D*—H⋯*A*
C6—H6⋯O3^i^	0.93	2.60	3.163 (5)	119
C9—H9*A*⋯O2^ii^	0.96	2.70	3.403 (4)	131

Symmetry codes: (i) 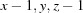; (ii) 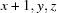.

**Fig. 1.**
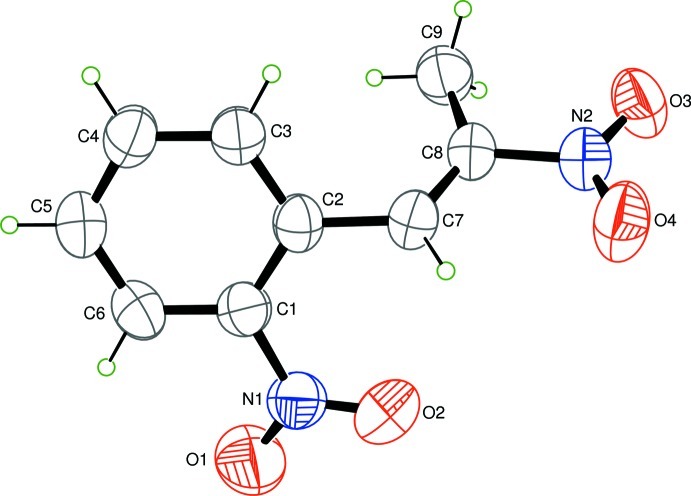
The asymmetric unit of the title compound with the atomic labeling scheme; displacement ellipsoids are drawn at the 50% probability level.

**Fig. 2.**
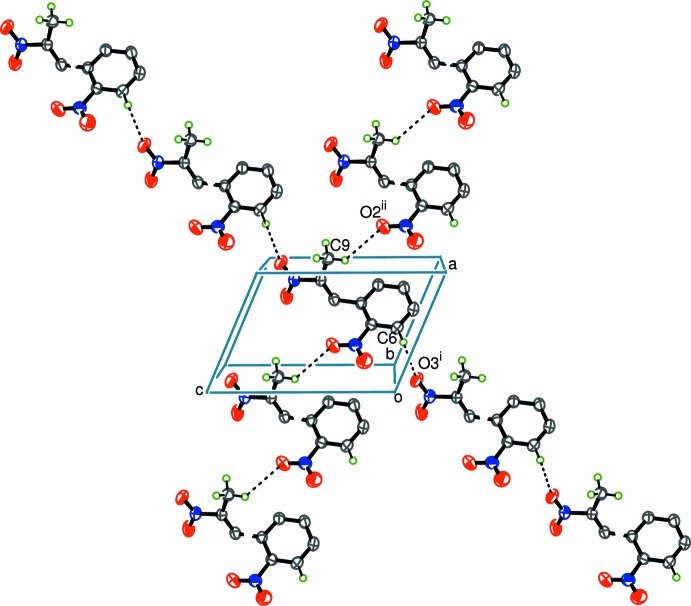
The view of intermolecular interactions illustrated as dash lines.

## supplementary crystallographic information

### Comment

Nitroalkenes are important organic intermediates, since they can be converted to synthetically useful N– and O-containing organic molecules, such as amines, aldehydes, carboxylic acids, or denitrated compounds (Ono, 2001; Berner *et al.*, 2002; Ballini & Petrini, 2004). As a contribution in this field, we have synthesized a series of nitroalkenes by employing benzaldehydes and nitroethane. We report here one of this nitroalkenes, *i.e.* the title compound. The C7═C8 bond involves the *E* configuration with the C3—C2—C7—C8 torsion angle of -57.7 (3)° (Fig. 1). The conformation of (I) is stabilized by weak intermolecular C6—H6···O3'and C9—H9A···O2' interactions (Fig. 2 and Table 1).

### Experimental

To a solution of 2-nitrobenzaldehyde (50 mmol) in AcOH (25 mL), nitroethane (75 mmol) was added, followed by butylamine (100 mmol, 7.4 mL). The mixture was sonicated at 60 °C, until GC showed full conversion of the aldehyde. The mixture was poured into ice water, the precipitate was filtered off, washed with water and recrystallized from EtOH/EtOAc to give the product. Single crystals were obtained by slow evaporation of a EtOH solution of the compound.

### Refinement

All H atoms were placed in calculated positions and refined using a riding model, with C—H = 0.93–0.96 Å, and with *U*_iso_(H) = 1.2 *U*_eq_(C) or 1.5 *U*_eq_(C) for methyl H atoms.

### Crystal data

**Table:**

C_9_H_8_N_2_O_4_	*F*(000) = 432
*M**_r_* = 208.17	*D*_x_ = 1.429 Mg m^−^^3^
Monoclinic, *P*2_1_/*n*	Mo *K*α radiation, λ = 0.71073 Å
Hall symbol: -P 2yn	Cell parameters from 5183 reflections
*a* = 6.8274 (9) Å	θ = 3.2–27.5°
*b* = 15.5666 (12) Å	µ = 0.12 mm^−^^1^
*c* = 9.9045 (10) Å	*T* = 296 K
β = 113.202 (3)°	Chunk, yellow
*V* = 967.51 (18) Å^3^	0.58 × 0.46 × 0.32 mm
*Z* = 4	

### Data collection

**Table:**

Rigaku R-AXIS RAPID/ZJUG diffractometer	1736 independent reflections
Radiation source: rotating anode	1193 reflections with *I* > 2σ(*I*)
Graphite monochromator	*R*_int_ = 0.072
Detector resolution: 10.00 pixels mm^-1^	θ_max_ = 25.2°, θ_min_ = 3.4°
ω scans	*h* = −7→8
Absorption correction: multi-scan (*ABSCOR*; Higashi, 1995)	*k* = −18→18
*T*_min_ = 0.932, *T*_max_ = 0.964	*l* = −11→11
7450 measured reflections	

### Refinement

**Table:**

Refinement on *F*^2^	Secondary atom site location: difference Fourier map
Least-squares matrix: full	Hydrogen site location: inferred from neighbouring sites
*R*[*F*^2^ > 2σ(*F*^2^)] = 0.059	H-atom parameters constrained
*wR*(*F*^2^) = 0.135	*w* = 1/[σ^2^(*F*_o_^2^) + (0.019*P*)^2^ + 0.5218*P*] where *P* = (*F*_o_^2^ + 2*F*_c_^2^)/3
*S* = 1.00	(Δ/σ)_max_ < 0.001
1736 reflections	Δρ_max_ = 0.26 e Å^−^^3^
138 parameters	Δρ_min_ = −0.21 e Å^−^^3^
0 restraints	Extinction correction: *SHELXL97* (Sheldrick, 2008), Fc^*^=kFc[1+0.001xFc^2^λ^3^/sin(2θ)]^-1/4^
Primary atom site location: structure-invariant direct methods	Extinction coefficient: 0.170 (12)

### Special details

**Table:**

Geometry. All e.s.d.'s (except the e.s.d. in the dihedral angle between two l.s. planes) are estimated using the full covariance matrix. The cell e.s.d.'s are taken into account individually in the estimation of e.s.d.'s in distances, angles and torsion angles; correlations between e.s.d.'s in cell parameters are only used when they are defined by crystal symmetry. An approximate (isotropic) treatment of cell e.s.d.'s is used for estimating e.s.d.'s involving l.s. planes.
Refinement. Refinement of *F*^2^ against ALL reflections. The weighted *R*-factor *wR* and goodness of fit *S* are based on *F*^2^, conventional *R*-factors *R* are based on *F*, with *F* set to zero for negative *F*^2^. The threshold expression of *F*^2^ > σ(*F*^2^) is used only for calculating *R*-factors(gt) *etc*. and is not relevant to the choice of reflections for refinement. *R*-factors based on *F*^2^ are statistically about twice as large as those based on *F*, and *R*- factors based on ALL data will be even larger.

### Fractional atomic coordinates and isotropic or equivalent isotropic displacement parameters (Å^2^)

**Table:**

	*x*	*y*	*z*	*U*_iso_*/*U*_eq_	
C1	0.5466 (4)	0.10207 (16)	0.2728 (3)	0.0619 (6)	
C2	0.7192 (4)	0.14601 (15)	0.3747 (3)	0.0601 (6)	
C3	0.8689 (4)	0.17747 (17)	0.3231 (3)	0.0709 (7)	
H3	0.9876	0.2069	0.3873	0.085*	
C4	0.8450 (5)	0.16585 (18)	0.1791 (3)	0.0774 (8)	
H4	0.9474	0.1873	0.1480	0.093*	
C5	0.6713 (5)	0.12290 (18)	0.0817 (3)	0.0782 (8)	
H5	0.6548	0.1159	−0.0154	0.094*	
C6	0.5215 (4)	0.09026 (17)	0.1286 (3)	0.0713 (7)	
H6	0.4042	0.0604	0.0637	0.086*	
C7	0.7489 (4)	0.16389 (15)	0.5287 (3)	0.0633 (7)	
H7	0.6399	0.1925	0.5440	0.076*	
C8	0.9185 (4)	0.14215 (15)	0.6453 (3)	0.0597 (6)	
C9	1.1075 (4)	0.09071 (19)	0.6571 (3)	0.0789 (8)	
H9A	1.0943	0.0736	0.5608	0.118*	
H9B	1.1164	0.0405	0.7156	0.118*	
H9C	1.2339	0.1247	0.7026	0.118*	
N1	0.3819 (4)	0.06343 (17)	0.3147 (3)	0.0799 (7)	
N2	0.9179 (4)	0.16809 (14)	0.7890 (2)	0.0702 (6)	
O1	0.2593 (4)	0.01245 (18)	0.2313 (3)	0.1285 (10)	
O2	0.3754 (4)	0.08268 (17)	0.4315 (3)	0.1097 (8)	
O3	1.0659 (3)	0.14555 (14)	0.8995 (2)	0.0925 (7)	
O4	0.7722 (4)	0.21184 (15)	0.7936 (2)	0.1023 (8)	

### Atomic displacement parameters (Å^2^)

**Table:**

	*U*^11^	*U*^22^	*U*^33^	*U*^12^	*U*^13^	*U*^23^
C1	0.0613 (14)	0.0631 (15)	0.0609 (15)	0.0036 (12)	0.0238 (12)	0.0103 (11)
C2	0.0654 (14)	0.0611 (14)	0.0548 (13)	0.0078 (12)	0.0248 (12)	0.0053 (11)
C3	0.0736 (16)	0.0778 (17)	0.0626 (16)	−0.0069 (14)	0.0282 (13)	−0.0023 (13)
C4	0.0854 (18)	0.089 (2)	0.0661 (16)	−0.0078 (15)	0.0387 (15)	0.0021 (14)
C5	0.093 (2)	0.0868 (19)	0.0555 (15)	0.0000 (16)	0.0296 (15)	0.0035 (13)
C6	0.0718 (16)	0.0734 (17)	0.0591 (16)	0.0003 (13)	0.0155 (13)	0.0031 (13)
C7	0.0715 (15)	0.0649 (15)	0.0604 (15)	0.0069 (12)	0.0335 (13)	0.0019 (12)
C8	0.0699 (15)	0.0588 (14)	0.0530 (14)	−0.0005 (12)	0.0271 (12)	−0.0022 (11)
C9	0.0728 (17)	0.089 (2)	0.0725 (17)	0.0113 (14)	0.0258 (14)	−0.0033 (14)
N1	0.0720 (15)	0.0871 (17)	0.0793 (17)	−0.0004 (13)	0.0284 (13)	0.0154 (13)
N2	0.0839 (15)	0.0681 (14)	0.0606 (13)	0.0013 (12)	0.0305 (12)	−0.0016 (10)
O1	0.1249 (18)	0.146 (2)	0.1092 (19)	−0.0697 (18)	0.0400 (15)	−0.0081 (16)
O2	0.1001 (16)	0.144 (2)	0.1069 (18)	−0.0120 (14)	0.0642 (14)	−0.0012 (15)
O3	0.0962 (14)	0.1094 (16)	0.0554 (11)	0.0055 (12)	0.0120 (10)	−0.0029 (10)
O4	0.1208 (17)	0.1218 (18)	0.0747 (13)	0.0400 (15)	0.0497 (13)	0.0037 (12)

### Geometric parameters (Å, º)

**Table:**

C1—C6	1.382 (3)	C7—C8	1.318 (3)
C1—C2	1.392 (3)	C7—H7	0.9300
C1—N1	1.472 (3)	C8—C9	1.483 (3)
C2—C3	1.400 (3)	C8—N2	1.481 (3)
C2—C7	1.483 (3)	C9—H9A	0.9600
C3—C4	1.381 (3)	C9—H9B	0.9600
C3—H3	0.9300	C9—H9C	0.9600
C4—C5	1.372 (4)	N1—O1	1.212 (3)
C4—H4	0.9300	N1—O2	1.212 (3)
C5—C6	1.376 (4)	N2—O3	1.212 (3)
C5—H5	0.9300	N2—O4	1.221 (3)
C6—H6	0.9300		
			
C6—C1—C2	122.6 (2)	C8—C7—C2	124.8 (2)
C6—C1—N1	116.1 (2)	C8—C7—H7	117.6
C2—C1—N1	121.3 (2)	C2—C7—H7	117.6
C3—C2—C1	116.0 (2)	C7—C8—C9	130.1 (2)
C3—C2—C7	119.1 (2)	C7—C8—N2	116.0 (2)
C1—C2—C7	124.8 (2)	C9—C8—N2	113.8 (2)
C4—C3—C2	121.7 (2)	C8—C9—H9A	109.5
C4—C3—H3	119.2	C8—C9—H9B	109.5
C2—C3—H3	119.2	H9A—C9—H9B	109.5
C5—C4—C3	120.5 (3)	C8—C9—H9C	109.5
C5—C4—H4	119.8	H9A—C9—H9C	109.5
C3—C4—H4	119.8	H9B—C9—H9C	109.5
C6—C5—C4	119.6 (2)	O1—N1—O2	122.5 (3)
C6—C5—H5	120.2	O1—N1—C1	118.3 (3)
C4—C5—H5	120.2	O2—N1—C1	119.1 (3)
C5—C6—C1	119.6 (2)	O3—N2—O4	122.0 (2)
C5—C6—H6	120.2	O3—N2—C8	118.1 (2)
C1—C6—H6	120.2	O4—N2—C8	119.9 (2)
			
C6—C1—C2—C3	−0.5 (4)	C1—C2—C7—C8	124.7 (3)
N1—C1—C2—C3	178.0 (2)	C2—C7—C8—C9	−5.5 (4)
C6—C1—C2—C7	177.1 (2)	C2—C7—C8—N2	178.6 (2)
N1—C1—C2—C7	−4.4 (4)	C6—C1—N1—O1	12.6 (4)
C1—C2—C3—C4	0.4 (4)	C2—C1—N1—O1	−166.0 (3)
C7—C2—C3—C4	−177.3 (2)	C6—C1—N1—O2	−168.4 (2)
C2—C3—C4—C5	0.2 (4)	C2—C1—N1—O2	13.1 (4)
C3—C4—C5—C6	−0.9 (4)	C7—C8—N2—O3	176.3 (2)
C4—C5—C6—C1	0.8 (4)	C9—C8—N2—O3	−0.3 (3)
C2—C1—C6—C5	−0.1 (4)	C7—C8—N2—O4	−4.6 (3)
N1—C1—C6—C5	−178.6 (2)	C9—C8—N2—O4	178.8 (2)
C3—C2—C7—C8	−57.7 (3)		

### Hydrogen-bond geometry (Å, º)

**Table:**

*D*—H···*A*	*D*—H	H···*A*	*D*···*A*	*D*—H···*A*
C6—H6···O3^i^	0.93	2.60	3.163 (5)	119
C9—H9*A*···O2^ii^	0.96	2.70	3.403 (4)	131

Symmetry codes: (i) *x*−1, *y*, *z*−1; (ii) *x*+1, *y*, *z*.
